# Carotenoids, Vitamin C, and Antioxidant Capacity in the Peel of Mandarin Fruit in Relation to the Susceptibility to Chilling Injury during Postharvest Cold Storage

**DOI:** 10.3390/antiox9121296

**Published:** 2020-12-17

**Authors:** Florencia Rey, Lorenzo Zacarías, María J. Rodrigo

**Affiliations:** Department of Food Biotechnology, Institute of Agrochemistry and Food Technology (IATA-CSIC), 46980 Valencia, Spain; floreyrob@iata.csic.es (F.R.); lzacarias@iata.csic.es (L.Z.)

**Keywords:** antioxidant capacity, chilling injury, citrus fruit, carotenoids, mandarin, vitamin C

## Abstract

Chilling injury (CI) is a postharvest disorder occurring in the fruit of cold-sensitive *Citrus* species during storage at low temperatures. This study investigated the involvement of carotenoids and vitamin C, two major antioxidants of citrus peel, and the antioxidant capacity in the CI susceptibility of mandarin fruit. To that end, the fruit of three commercial varieties, Fortune, Nova, and Nadorcott, with significant differences in CI susceptibility, were selected. By on-tree fruit bagging, carotenoids and vitamin C contents were modified, and a differential effect of each cultivar on CI was observed. Carotenoid analysis in the peel revealed a strong negative correlation between total carotenoid concentration (TCC) at harvest, and specifically of β-cryptoxanthin and violaxanthin, and CI index at the end of storage. In contrast, vitamin C content was significantly and positively correlated with CI susceptibility. The antioxidant activity assessed by the DPPH• and FRAP reflected the contribution of vitamin C to the antioxidant system, while the SOAC assay correlated positively with TTC, β-cryptoxanthin, and violaxanthin. Collectively, the antioxidant capacity of carotenoids at harvest, as efficient singlet oxygen quenchers, suggests a protective role against the development of CI in mandarin fruit, while vitamin C is not likely playing a critical role.

## 1. Introduction

Storage at low temperatures is the technology most widely used to maintain fruit quality and extend postharvest life. However, prolonged storage at low temperature may cause physiological disorders in many species of tropical and subtropical origin, such as citrus fruit, that are prone to develop external injuries during cold storage, depreciating their quality and increasing postharvest losses. Furthermore, some countries enforce quarantine measures on citrus fruit imports, requiring storage during transport at 1–2 °C. Damage developed during storage at low non-freezing temperatures is commonly known as chilling injury (CI) and, even though macroscopic symptoms in *Citrus* fruit vary between species and varieties, they are generally manifested as brown pit-like depressions in the flavedo (outer layer of fruit peel) that expand progressively over the peel surface and become darker under prolonged exposure to low temperatures [1,2,3].

Many pre- and postharvest factors have been described as influencing the susceptibility of citrus fruit to CI during postharvest storage. The genotype of the species/variety is an intrinsic genetic factor determining CI sensitivity or tolerance. Among *Citrus* species, lemon and grapefruit are more sensitive to CI than orange and mandarin, which are generally more tolerant [3]. Nonetheless, within mandarins and their hybrids, there is great variability in susceptibility to CI, with cultivars ranging from tolerant, such as those of the Clementine group, to highly sensitive, such as the hybrid Fortune [2,3]. Environmental factors and agronomic practices, such as temperature before harvest, maturity stage, or canopy position, among others, can affect the biochemical profiling of the fruit peel and their response to postharvest cold storage and sensitivity to CI [3,4,5]. In line with this, higher carbohydrate content has been associated with higher tolerance to disorders during cold storage of the Nules Clementine [6,7] and Pinalate sweet orange [8], although no relationship between changes in carbohydrate content and CI could be established in Fortune mandarin [9]. Other peel properties, such as rind coloration, are also influenced by environmental and endogenous factors and may be involved in the differential sensitivity to CI among *Citrus* cultivars [5,6].

The coloration of mature citrus fruit is mainly due to the accumulation of carotenoids [10,11]. Light is one of the most crucial environmental factors influencing carotenoid biosynthesis [12,13,14] and, in general, stimulates carotenoid accumulation by up-regulating key genes of their biosynthetic pathway [14,15,16]. In citrus fruit, light can have the opposite effect according to the tissue or species. For instance, the fruit of the Nules Clementine mandarin and Navelina orange, which were bagged on the tree or harvested from inside of the tree canopy (reduced light incidence), developed a paler rind color than those exposed to normal light incidence, which had an intense orange coloration and accumulated higher contents of total carotenoids [4,6,7,14]. The opposite effect was found in the fruit of the Star Ruby grapefruit, where avoiding light during fruit development on the tree promoted the accumulation of carotenes, in particular the red carotene lycopene, resulting in fruit with an intense red coloration of the peel in comparison with the pale-yellow peel of those exposed to direct sunshine [17].

The influence of carotenoid content and composition on the tolerance of citrus fruit to CI has been studied in grapefruit, where white cultivars accumulating very low levels of carotenoids in the peel were more prone to develop CI than red grapefruit with moderate to high levels [5]. Moreover, symptoms of CI in red grapefruit, such as the Star Ruby, were restricted to the yellow areas of the peel, whereas the red areas accumulating high amounts of lycopene were undamaged [5,17]. In mandarin fruit, the involvement of carotenoids on CI has not been studied in depth. However, in Clementine mandarin under South African growing conditions, it has been detected that fruit borne inside the tree canopy are less colored, contain less carotenoid content, and are more prone to develop rind-breakdown when stored at low temperature than fruits from outside the canopy [6,7].

The potential role of carotenoids in conferring higher tolerance to CI has been associated with their properties as antioxidants. Oxidative stress is thought to be a secondary response of plant cells to CI caused by an increase in the production of reactive oxygen species (ROS) due to low-temperature stress and the plant’s inability to counteract this proliferation of ROS [3,18]. Among carotenoids, lycopene is reported as an efficient singlet oxygen quencher [19,20]. Accumulation of this carotenoid in the flavedo of CI-tolerant red grapefruit was associated with lower oxidative damage (membrane structure and lipid peroxidation), higher singlet oxygen scavenging capacity (SOAC), and higher catalase activity [21]. Nonetheless, the potential role of other carotenoids as antioxidants, which accumulate at important concentrations in the peel of mandarins, in the tolerance/sensitivity of these fruits to CI has not been explored in detail yet.

Ascorbic acid (vitamin C) is another major antioxidant compound of the citrus fruit peel, which contributes to the antioxidant capacity of plant cells by detoxifying ROS and free radicals [22,23,24]. Vitamin C content in citrus varies among different species and cultivars and is widely affected by many pre-harvest factors, of which light is one of the most noteworthy [25,26,27]. Light avoidance produced a dramatic reduction in the content of vitamin C in the peel of orange, mandarin, and grapefruit, and downregulated the expression of key genes involved in vitamin C synthesis and recycling [7,17,28]. The relation between vitamin C content and tolerance to CI in citrus fruit has been explored with controversial results. While a direct link between CI incidence and high vitamin C concentration was observed in the Navel orange [29], an indirect relationship was reported in Clementine mandarins [30], and no relation could be established in the Valencia orange [31] or Star Ruby grapefruit [32]. However, higher levels of ascorbic acid were found in the peel of CI-sensitive Star Ruby grapefruit than in CI-tolerant, at harvest and after storage at 2 °C [21]. It has been hypothesized that abiotic stress conditions, inducing ROS production, increase the requirements and response of plant tissues for antioxidants compounds, and this could explain the rise in ascorbic acid contents in CI-sensitive fruit [21,33].

Despite previous evidence indicating a relationship between sensitivity to CI and antioxidant compounds in fruits of different *Citrus* species and varieties, systematic research of this potential relation in mandarin fruit is still lacking. Therefore, the aim of the current study was to investigate the involvement of carotenoids and vitamin C, and the changes in antioxidant activity/capacity, in the susceptibility of mandarin fruit to CI. To that end, we used the fruit of three commercial varieties: Fortune, Nova, and Nadorcott, with differences in peel pigmentation and in the susceptibility to develop CI during postharvest cold storage.

## 2. Materials and Methods

### 2.1. Plant Material and Storage Conditions

Fruit from 3 mandarin cultivars was used in this work: Fortune (*Citrus clementina* Hort. Ex Tan. × *Citrus reticulata* Blanco), Nova (*Citrus clementina* Hort. ex Tanaka × (*Citrus paradisi* Mc-Fadyen × *Citrus reticulata* Blanco)) and Nadorcott (*Citrus reticulata* Blanco). Fruits were harvested from the Citrus Germplasm Bank (Instituto Valenciano de Investigaciones Agrarias, IVIA, Valencia, Spain) and commercial orchards located in Valencia (Spain) under similar environmental conditions and agronomic practices. In the 3 cultivars, fruit bagging experiments were also performed, and light incidence was avoided during fruit ripening by covering the fruits with black polyethylene bags at the mature green stage (covered fruit, C), following the methodology previously described by Lado et al. [14]. Control fruit (non-covered, NC) were located outside of the canopy and exposed to direct natural light and normal photoperiodic conditions. At least 50 fruit were used for each treatment. At commercial maturity, both NC and C fruit were harvested and stored at 2 °C and 85% RH for up to 8 weeks. At harvest and periodically through cold storage, samples of flavedo (outer colored layer of citrus fruit peel) were excised, frozen in liquid nitrogen, ground to a fine powder, and stored at −80 °C until analysis.

### 2.2. Chilling Injury Evaluation

Fruit from each cultivar was inspected for CI symptoms throughout cold storage. Chilling injury in mandarin fruit generally manifested as small brown pit-like depressions in the flavedo (‘pitting’), which became darker and sunken and expanded over the fruit surfaced with increased exposure to low temperatures [2,3]. According to the severity and extension of CI symptoms, mandarin fruits were assessed visually and classified using the following scale: 0 = no pitting; 1 = small pits covering <25% of the fruit surface; 2 = darker pits covering between 25 and 50% of the surface; and 3 = severe damage covering 50 to 100% of the surface (Figure S1). Results were expressed as CI index, which was calculated by adding the product of the number of fruit in each category multiplied by the score for each category and afterward dividing this amount by the total number of fruit evaluated [34]. For CI evaluation, at least 30 fruit per treatment and cultivar were used.

### 2.3. External Peel Color Evaluation

Peel color was measured during cold storage using a Minolta CR-400 colorimeter (Minolta, Osaka, Japan) on 3 areas of the equatorial plane of the fruit and expressed as the *a/b* Hunter ratio [35]. The *a/b* ratio was negative for green fruit, around zero for yellow fruit at the color break, and positive for orange and red colorations. For color evaluation, at least 10 fruit per treatment and cultivar were used.

### 2.4. Carotenoid Extraction and Analysis

Carotenoids were extracted from the flavedo as described previously in Rodrigo et al. [35], with slight modifications. Briefly, 0.5 g of flavedo tissue were extracted with 3 mL of methanol (HPLC grade, Sharlau, Barcelona, Spain), 1.5 mL Tris buffer (50 mM Tris-HCl pH 7.5 with 1 M NaCl) and 4 mL of chloroform (HPLC grade, Sharlau, Barcelona, Spain) in a mortar and pestle with sea sand (PanReac AppliChem, Barcelona, Spain) as an abrasive. The homogenate was recovered in a polypropylene tube and sonicated in a XUBA3 ultrasonic water bath (Grant Instruments, Cambridge, England) for 5 min at room temperature. After this, samples were centrifuged at 4500× *g* for 10 min at 4 °C for liquid-phase separation. The hypophase was recovered in a new tube and the aqueous phase was re-extracted with chloroform until it was colorless. The pooled chloroform extracts were concentrated on a rotatory evaporator at 30 °C, and saponified overnight using a 10% methanolic:KOH solution under N_2_ atmosphere. Saponified carotenoids were recovered from the upper phase after adding water and petroleum ether:diethyl ether (9:1, *v*:*v*) to the mixture. Extracts were dried under a nitrogen stream and kept at −20 °C until further analysis.

Carotenoid composition was analyzed by high-performance liquid chromatography (HPLC) with a waters liquid chromatography system equipped with a 600E pump, coupled to a 2998 photodiode array detector (PAD) and Empower3 software (Waters, Barcelona, Spain). A C30 carotenoid column (250 × 4.6 mm, 5 µm) coupled to a C30 guard column (20 × 4.0 mm, 5 µm) (YMC, Tecknochroma, Barcelona, Spain) was used. Samples were prepared for HPLC by dissolving the dried carotenoid extracts in chloroform:methanol:acetone (3:2:1, *v*:*v*:*v*). A ternary gradient elution with methanol, water, and methyl tert-butyl ether was used for carotenoid separation as reported in previous work [35]. The PAD was set to scan from 250 to 540 nm throughout the elution profile. For each elution, a Maxplot chromatogram was obtained, which integrated each carotenoid peak at its corresponding maximum absorbance wavelength. Individual carotenoid content was calculated using previously described calibration curves [14,36]. A characteristic chromatogram is displayed in Figure S2. The limit of detection and working linear range for carotenoids quantified is shown in Table S1. All operations were carried out on ice under dim light to prevent photodegradation, isomerization, and structural changes of carotenoids. At least 2 independent flavedo extracts were obtained for each sample, and each extract was injected twice into the HPLC system. Results were the mean of at least 2 replicates from each extract (mean ± SD). Total carotenoids (TCC) were calculated as the sum of all the individual carotenoids quantified. Standard deviations were calculated from the mean of the total carotenoids value of each replicate. The concentrations are expressed as µg per g of fresh weight (FW).

### 2.5. Vitamin C Determination

Ascorbic acid (AsA) and dehydroascorbic acid (DHA) contents were determined as described by Alós et al. [26]. Briefly, 0.5 g of flavedo tissue was homogenized for 1 min using a homogenizer (Polytron, Eschbach, Germany) with 0.1% metaphosphoric acid (4 mL) at maximum speed. The homogenate was centrifuged for 10 min at 4500× *g* at 4 °C. Then, the supernatant was filtered through a C18 cartridge (SepPak, Waters, Barcelona, Spain) previously activated with 4 mL of methanol, 4 mL of water, and 4 mL of 0.1% metaphosphoric acid. The filtered extract was re-filtered through a 0.45 µm nylon filter (25 mm diameter, Análisis Vínicos, Tomelloso, Spain), and the filtrate was injected in the HPLC-PAD system for AsA determination. DHA content was calculated from the difference between total vitamin C and the AsA content. To determine total vitamin C, an adaptation of the protocol described by Davey et al. [37] was used. Thus, 200 µL aliquot of the above-mentioned filtrate was incubated for 15 min at room temperature with 100 µL 200 mM DTT in 400 mM Tris-base, which generated a final pH of 6–6.8. Then, the reaction was stopped by acidification with 100 µL of 8.5% orthophosphoric acid.

For AsA and total vitamin C determination, 10 µL of the above mentioned extracts were injected in a Dionex HPLC system with a PAD and Chromeleon software (Dionex, Thermo Fisher Scientific, Barcelona, Spain), an Ultrabase C18 column (100 × 4.6 mm, 2.5 µm) with a mobile phase of methanol:water pH 2.5 (adjusted with metaphosphoric acid, 15:85, *v*:*v*) at 0.2 mL min^−1^ flux. The temperature of the column was set at 35 °C. The PAD was set to monitor the spectrum from 200 to 450 nm, and the peak area at 248 nm (wavelength of maximum absorption for AsA) was used for quantification. A characteristic chromatogram was displayed in Figure S3. The method was calibrated daily with a curve of an AsA standard solution in 0.1% metaphosphoric acid. Results were expressed as mg per 100 g of sample (FW).

### 2.6. Antioxidants Assays

#### 2.6.1. DPPH and FRAP Assays

Two different assays were performed to measure antioxidant activity in the flavedo: (i) Reaction with 2,2-diphenyl-1-picrylhydrazyl (DPPH• assay) and (ii) ferric-reducing antioxidant power in aqueous solution (FRAP assay). The same extraction method was carried out for both assays. Briefly, 0.15 g of frozen flavedo tissue was extracted in 3 mL of 80% methanol using a pre-chilled mortar and pestle on an ice bath. The homogenate was centrifuged for 5 min at 4500× *g* (4 °C), and the collected supernatant was immediately used for analysis. Each assay was replicated twice, and a curve of AsA solution in methanol 80% was used as a standard in both assays.

Determination of the DPPH• assay was performed as described in Parra-Rivero et al. [38] with slight modifications. In a 96-well microplate, 10 µL of the sample extract were mixed with 290 µL DPPH• methanolic solution (100 µM) and allowed to stand for 30 min at room temperature in complete darkness. Each sample was replicated in 3 wells within one microplate, and methanol (80%) was used as a control (10 µL methanol 80% + 290 µL of DPPH•). Changes in the absorbance were measured at 512 nm in a UV/Vis microplate spectrophotometer (Multiskan FC, Thermo Fisher Scientific, Barcelona, Spain) and compared to the control. DPPH• scavenging capacity was expressed as inhibition percentages by the Formula (1):

% DPPH• scavenging capacity = [(^515 nm^Acontrol − ^515 nm^Asample)/^515 nm^Acontrol] × 100 (1)

For the FRAP assay, the procedure described by Parra-Rivero et al. [38] adapted for flavedo tissue was followed. First, a reagent was prepared with 2,4,6-tri(2-pyridyl)-1,3,5-triazine (10 mM), acetate buffer (300 mM) and a ferric chloride solution (20 mM) (1:10:1, *v*:*v*:*v*). Then, 40 µL sample extract was mixed with 260 µL of the FRAP reagent on a microplate well, allowing the microplate to stand at 37 °C for 30 min after loading. Each sample was replicated in 3 wells within 1 microplate and methanol (80%) was used for control (40 µL methanol 80% + 260 µL of FRAP). Absorbance was measured at 593 nm in a UV/Vis microplate spectrophotometer (Multiskan FC, Thermo Fisher Scientific, Barcelona, Spain). To calculate FRAP, the antioxidant activity sample absorbance was first corrected by the absorbance of control. In both assays, results were the mean of the replicates in the 2 microplates (mean ± SE).

#### 2.6.2. Singlet Oxygen Absorption Capacity (SOAC)

The SOAC determination was done following previous procedures [21,39,40] with slight modifications. Briefly, 0.3 g of frozen flavedo tissue was extracted in 6 mL of cooled ethanol:chloroform:water (50:50:1, *v*:*v*:*v*) using a pre-chilled mortar and pestle on an ice bath with sea sand (PanReac AppliChem, Barcelona, Spain) as an abrasive. Then, the homogenate was centrifuged for 5 min at 4500× *g* at 4 °C, and the collected supernatant was immediately used for analysis.

To measure the extracts ^1^O_2_ quenching capacity a competition reaction was carried out using endoperoxide (EP, Invitrotech, Kyoto, Japan) as a singlet oxygen generator and 2,5-diphenyl- 3,4-benzofuran (DPBF, Sigma–Aldrich, Barcelona, Spain) as an UV-Vis absorption probe in a 96-well quartz glass microplate. In each well, 15 µL of the peel extract was mixed with 150 µL of DPBF (0.8 mM solution) and 75 µL of EP (1 mM). The microplate was loaded in dim light and on an ice bath. Absorbance changes of DPBF at 413 nm were monitored during a 90 min reaction at 35 °C using a UV-Vis spectrophotometer microplate reader (SPECTROstar^®^ Omega, BMG Labtech, Offenburg, Germany). α-tocopherol (Sigma–Aldrich, Barcelona, Spain) was used as a standard compound and ethanol:chloroform:water (50:50:1, *v*:*v*:*v*) as a blank. The relative SOAC value for each sample was calculated with the following Formula (2):

(t1/2 sample − t1/2 blank)/(t1/2 α-toc − t1/2 blank) × ([α-toc, g L^−1^]/[sample, g L^−1^]) (2)

Each sample was replicated in 3 wells, and the assay was replicated twice. Results were the mean of the replicates in the 2 microplates (mean ± SE).

### 2.7. Statistical Analyses

Results were expressed as the mean ± standard error (SE). Unpaired Student’s t-test was used to determine the mean differences statistically between NC and C fruit, within each cultivar and sample date (significance level at *p* ≤ 0.05 or *p* ≤ 0.10). Additionally, a one-way ANOVA was carried out, and Tukey’s test (significance level at *p* ≤ 0.05) was used for mean comparisons among dates. The correlations between variables were examined by Pearson’s correlation. Analysis was made using the XLSTAT Software version 2019.3.2 (Addinsoft, Paris, France) and SigmaPlot version 14.0 (Systat Software, San Jose, CA, USA).

## 3. Results

### 3.1. Incidence of Chilling Injury and Changes in Peel Pigmentation of Fortune, Nova and Nadorcott Mandarins during Cold Storage

The mandarins Fortune, Nova, and Nadorcott were selected on their reported different sensitivity to CI and peel color intensity. Fortune and to a lower extent Nova fruit are prone to develop CI during storage at low temperatures [34,41], while Nadorcott appears to be more resistant to CI, even though it is susceptible to other postharvest blemishes [2,42]. Nadorcott is a late-maturing cultivar characterized by a deep reddish-orange rind and pinkish albedo, and excellent internal quality [43].

**Figure 1:**
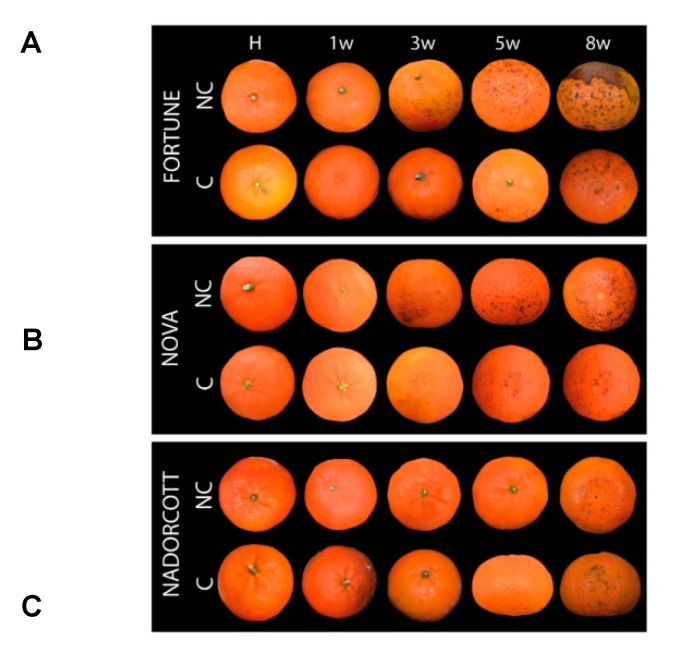
External appearance of fruit of Fortune (**A**), Nova (**B**), and Nadorcott (**C**) at harvest (H) and evolution of chilling injury (CI) symptoms during storage at 2 °C of non-covered (NC) and covered (C) fruit for up to 8 weeks.

**Figure 2:**
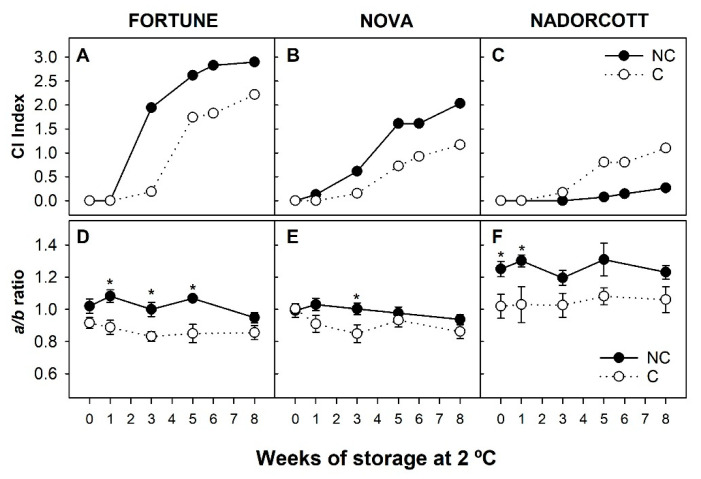
Development of chilling damage expressed as the CI index of non-covered (NC) and covered (C) fruit of Fortune (**A**), Nova (**B**), and Nadorcott (**C**); and rind color expressed as *a/b* ratio of non-covered (NC) and covered (C) fruit of Fortune (**D**), Nova (**E**), and Nadorcott (**F**), at harvest (0) and during storage at 2 °C. Asterisks indicate significant differences between NC and C fruit for each cultivar and sample date (*p* ≤ 0.05, *t*-test).

Sensitivity to CI was evaluated in NC and C fruit of Fortune, Nova, and Nadorcott during storage at 2 °C for up to 8 weeks (Figure 1 and Figure 2). Symptoms of CI appeared earlier in Fortune than in Nova mandarins, but in both cultivars, symptoms manifested as the typical peel pitting, with small shrunken darks pits spread over the surface that progressively expanded, creating large necrotic and depressed areas, depreciating external fruit quality (Figure 1 and Figure S1). In the fruit of the Nadorcott mandarin, CI symptoms were almost absent in control fruits, and only a few pits, in general around the stylar zone, were observed after prolonged cold storage (Figure 1 and Figure S4).

The CI index increased markedly in NC fruits of Fortune after 2 weeks of storage, reaching values of 2 by week 3 and about 3 after 5 weeks, and almost all fruit exhibited severe symptoms at the end of storage (Figure 2A). In Nova mandarin (NC fruit), CI increased steadily to reach at the end of the storage period an index of 2 (Figure 2B). NC fruits of Nadorcott mandarin were resistant to CI, developing an index of 0.3 at the end of the storage period (Figure 2C). Since light avoidance during the last stage of fruit development has been shown to induce cold-tolerance in Star Ruby grapefruit during postharvest storage [21], mature-green fruits of the three cultivars were covered, and the incidence of CI during cold storage evaluated. Covered fruit of Fortune and Nova mandarin exhibited a similar response during cold storage, with a delay in the development of symptoms and a reduction in CI at the end of the storage period with respect to NC fruits (Figure 2A,B). In contrast, light avoidance produced the opposite effect on the fruit of the Nadorcott mandarin, as the CI index was higher in NC fruit than in C after 3 weeks, being about 4-times higher at the end of the cold storage (Figure 2C).

Rind color (*a/b* Hunter ratio) was monitored in NC and C fruit of the three cultivars at harvest and during cold storage (Figure 2D–F). At harvest, NC fruits of the Nadorcott mandarin had a more intense coloration and higher *a/b* ratio than Fortune and Nova mandarins. Only C fruit of Nadorcott was affected by light avoidance, resulting in less colored fruit and a lower *a/b* ratio than NC fruit, whereas no significant differences were detected in Fortune and Nova. In general, during cold storage, the color index remained steady or decreased slightly and, although not always significant, C fruit were paler and had a lower *a/b* ratio than NC fruit (Figure 2D–F).

### 3.2. Changes in Carotenoids and Vitamin C in the Peel of Fortune, Nova, and Nadorcott Mandarins during Cold Storage

#### 3.2.1. Total Carotenoid Content and Carotenoid Composition

Carotenoid content and composition were analyzed in NC and C fruit of Fortune, Nova, and Nadorcott at harvest and after 3 and 8 weeks of cold storage (Table 1, Table 2 and Table 3). At harvest, the highest TCC was detected in the flavedo of Nadorcott NC fruit (582.20 ± 22.85 µg g^−1^ FW), which was 3.1- and 1.3-times higher than that of Fortune and Nova, respectively. Fruit bagging had a differential effect on TCC depending on the cultivar. In the peel of Fortune mandarin, TCC was lower than in the other two cultivars and was not affected by the absence of light. During cold storage, TCC increased in NC fruit and remained constant in C fruit of Fortune (Table 1). Light avoidance reduced by about 30% TCC in Nadorcott and Nova mandarins with respect to NC fruit exposed to natural photoperiodic conditions (Table 2 and Table 3). Cold storage did not affect TCC in NC and C fruit of the Nova mandarin (Table 2) but provoked a reduction in NC fruit of Nadorcott (Table 3). Nonetheless, TCC at the end of cold storage remained higher in NC than in C fruits of these two mandarins (Table 2 and Table 3).

By HPLC-PAD analysis, a total of 22 carotenoid-like peaks were identified in the flavedo of the three mandarins (Table S2). Comparing the spectra and retention times of these peaks to authentic standards or those described in other works using similar chromatographic conditions [14,35,44], we were able to unambiguously identify 13 carotenoids (Table S2), which represented more than 90% of TCC (Table 1, Table 2 and Table 3). The flavedo of the three mandarin cultivars displayed a carotenoid profile characteristic of mature mandarin, accumulating predominantly β,β-xanthophylls, which represented 60–80% of TCC. The β,β-xanthophyll violaxanthin and, in particular, its 9-Z isomer, was the main carotenoid accounting for 40–60% of TCC. The content of individual carotenoids differed among the three cultivars, and, in general, the highest concentrations for all carotenoids were detected in Nadorcott. However, Nova showed the highest content of the colorless carotenes phytoene and phytofluene in all samples. In the three cultivars, high concentrations of the *Citrus*-specific C30 apocarotenoid β-citraurin, which confers an orange-reddish coloration, were quantified. At harvest, β-citraurin contents ranged from nearly 30 µg g^−1^ in Fortune to around 58 µg g^−1^ FW in Nova (Table 1, Table 2 and Table 3). The concentration of β-cryptoxanthin, the characteristic xanthophyll of mandarins, which also confers a deeper orange coloration to the peel, was extremely high in Nadorcott (~65 µg g^−1^) when compared to the other cultivars. In Nova, β-cryptoxanthin content was also high (between 20–25 µg g^−1^), while in Fortune, moderate to low amounts (below 5 µg g^−1^) were detected.

When the carotenoids profile between NC and C fruit was compared in Nova and Nadorcott, the differences in TCC were explained by a higher accumulation of almost all carotenoids in NC fruits at harvest, which remained during storage (Table 2 and Table 3). The concentration of phytoene was two-fold higher in NC fruit compared to C fruit in most samples of Nova and Nadorcott. Violaxanthin content was 20% higher in NC than in C fruit of Nova and Nadorcott at harvest and, while differences in Nova persisted during storage, in Nadorcott, they shortened, and no significant differences were detected after 8 weeks. The concentration of antheraxanthin was also higher in NC than C fruit of both Nova (2–3 times higher) and Nadorcott (about 1.5-fold). At harvest, the levels of β-cryptoxanthin were not significantly different between NC and C fruit of Nadorcott but were higher in NC fruit of Nova. Regarding β-citraurin, an apocarotenoid with high impact on the peel color, the content at harvest in Nova was higher in C than in NC fruit, whereas no differences were observed in Nadorcott. After 8 weeks of storage, β-citraurin levels were the same between NC and C fruit of Nova and significantly higher in NC fruit of Nadorcott than in C fruit. In Fortune mandarin, differences between NC and C fruit and changes in carotenoid levels during storage differed from those described in the other two cultivars. At harvest, the concentration of phytoene and phytofluene was significantly higher in C fruit. However, after 8 weeks of cold storage, the opposite effect occurred, and NC fruit accumulated higher phytoene. Moreover, differences in individual carotenoids between NC and C Fortune fruit during cold storage were less evident than for Nova and Nadorcott, being remarkable the lower concentration of phytoene, β-cryptoxanthin, β-citraurin, and other β,β-xanthophylls in C fruits after 8 weeks of storage (Table 1).

#### 3.2.2. Vitamin C Content

**Figure 3:**
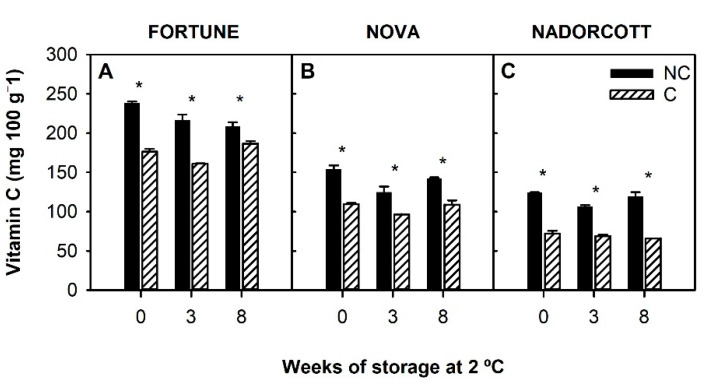
Total vitamin C content (mg 100 g^−1^) at harvest (0) and during storage at 2 °C in the peel of non-covered (NC) and covered (C) fruit of Fortune (**A**), Nova (**B**), and Nadorcott (**C**). Asterisk indicates significant differences between NC and C fruit for each cultivar and sample date (*p* ≤ 0.05, *t*-test).

Vitamin C (AsA + DHA) content was determined in the flavedo of NC and C fruit of the three mandarins at harvest and after 3 and 8 weeks of cold storage (Figure 3). Among NC fruit, Fortune accumulated the highest amount of vitamin C (~240 mg 100 g^−1^), which was twice the content detected in Nova and Nadorcott, both with similar levels (~120–150 mg 100 g^−1^). Light deprivation produced a significant reduction in vitamin C content in the flavedo of all varieties (around 50% in Nadorcott and 30% in Fortune and Nova). During cold storage, vitamin C remained nearly constant, and the differences between NC and C fruit were maintained.

### 3.3. Antioxidant Activity and Singlet Oxygen Absorption Capacity in the Peel of Fortune, Nova, and Nadorcott Mandarins during Cold Storage

**Figure 4:**
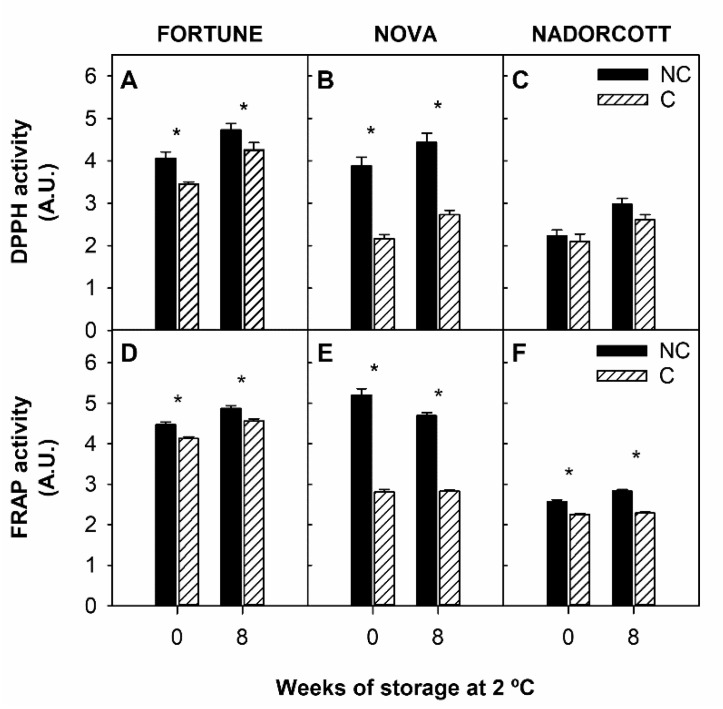
DPPH• antioxidant activity in the peel of non-covered (NC) and covered (C) fruit of Fortune (**A**), Nova (**B**), and Nadorcott (**C**); FRAP antioxidant activity in the peel of non-covered (NC) and covered (C) fruit of Fortune (**D**), Nova (**E**), and Nadorcott (**F**), at harvest (0) and after 8 weeks of storage at 2 °C. Asterisk indicates significant differences between NC and C fruit for each cultivar and sample date (*p* ≤ 0.05, *t*-test).

The antioxidant activity of the flavedo of the three mandarin cultivars was determined in hydrophilic extracts by DPPH• (radical scavenging activity) and FRAP (reducing activity) at harvest and after 8 weeks of storage in both NC and C fruit (Figure 4). Results obtained by both DPPH• and FRAP methods were similar. At harvest, the DPPH• and FRAP antioxidant activity of Fortune and Nova mandarin were comparable and about twice that of Nadorcott mandarin. Light avoidance produced a slight reduction in the antioxidant activity of Fortune and Nadorcott mandarins, and reduced between 35–45% that of Nova fruit. The DPPH• and FRAP antioxidant activity of the extracts remained fairly stable after 8 weeks of cold storage in both NC and C fruit of the three cultivars (Figure 4). Extracts from Fortune fruit (NC and C) and Nova NC fruit showed the highest relative values of DPPH• and FRAP antioxidant activity in comparison to Nadorcott (NC and C) and Nova C fruit, which showed 50 to 20% lower values, respectively (Figure 4).

**Figure 5:**
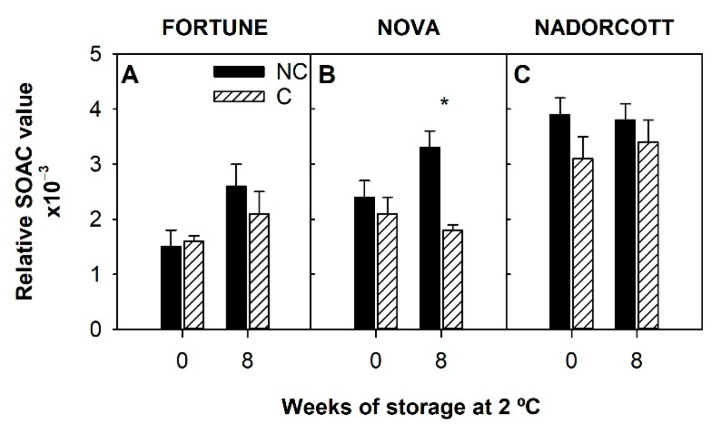
Relative SOAC value at harvest (0) and after 8 weeks of storage at 2 °C in the peel of non-covered (NC) and covered (C) fruit of Fortune (**A**), Nova (**B**), and Nadorcott (**C**). Asterisks indicate significant differences between NC and C fruit for each cultivar and sample date (*p* ≤ 0.05, *t*-test).

The antioxidant potential of the flavedo of the three cultivars at harvest and after 8 weeks of cold storage was also determined by the SOAC assay, which measures the singlet oxygen scavenging capacity of a lipophilic extract. In general, SOAC values were higher in Nadorcott fruit than in the other cultivars. At harvest, the SOAC capacity of NC Nadorcott fruit extract was 62% and 40% higher than that of Fortune and Nova, respectively, and these differences were slightly reduced after 8 weeks of storage (Figure 5). SOAC was not significantly affected by light deprivation, at harvest or after cold storage, with the exception of Nova after 8 weeks of storage when the SOAC was 1.8 times higher in NC than in C fruit (Figure 5B).

### 3.4. Correlation Analysis between Chilling Injury Index, Carotenoids, Vitamin C and DPPH•, FRAP and SOAC Antioxidant Activity/Capacity in the Peel of Fortune, Nova, and Nadorcott during Cold Storage

The correlation between the CI index after 8 weeks of cold storage, carotenoid content (total and individual), vitamin C, and DPPH•, FRAP and SOAC antioxidant activity/capacity at harvest were analyzed. The analysis integrated data from the three cultivars in both NC and C conditions. According to the data in Table 4, CI index after cold storage was significantly and negatively correlated with TCC (*r*^2^ = −0.81), β-cryptoxanthin (*r*^2^ = −0.82), violaxanthin (*r*^2^ = −0.85), and SOAC capacity (*r*^2^ = −0.88) at harvest. On the other hand, CI showed a significant positive correlation with vitamin C content (*r*^2^ = 0.82), DPPH• (*r*^2^ = 0.89) (sample’s reducing activity) and FRAP (*r*^2^ = 0.81) (sample’s radical scavenging activity). In addition, the correlation analysis shows that the DPPH• method reflects the vitamin C contribution to the antioxidant system (*r*^2^ = 0.87), and that SOAC is highly influenced by TCC (*r*^2^ = 0.96) and by the individual xanthophylls violaxanthin (*r*^2^ = 0.99) and β-cryptoxanthin (*r*^2^ = 0.92).

## 4. Discussion

Exposure of *Citrus* fruit to chilling temperatures during storage supposes a challenge in species sensitive to the development of CI symptoms, which depreciates their external quality and increases postharvest losses. Fruit conditions at harvest, including peel color, can influence cold sensitivity during postharvest [2,3]. The relationship between peel pigmentation and sensitivity to CI has been explored in grapefruit cultivars, showing that those with red-colored peel display higher tolerance to CI than yellow-colored ones [5,21]. In mandarin fruit, it has been traditionally considered that pale fruits from inside the tree canopy develop less external coloration and are more prone to postharvest blemishes, in particular CI, than fruits borne outside of the tree canopy with higher pigmentation [4,7,45]. However, solid evidence linking CI and carotenoids, or other antioxidants as vitamin C, in relation to their antioxidant activities are still lacking. To provide insights into this question, we selected fruit of three mandarin cultivars (Fortune, Nova, and Nadorcott) with marked differences in peel pigmentation and susceptibility to develop CI during cold storage. Since carotenoids and vitamin C contents in the peel of citrus fruit are largely influenced by light during ripening [14,17,25], pre-harvest bagging experiments were performed in order to modify the content of these compounds in the peel, and subsequently to study their effects on CI during storage.

Susceptibility to CI of the three mandarin cultivars during storage at 2 °C was markedly different (Figure 1 and Figure 2A–C and Figure S1). Fortune mandarin, as expected, was the most sensitive to CI. Nova mandarin was also chilling-sensitive but developed chilling symptoms at a lower rate than Fortune, in accordance with previous studies [3,41]. Nadorcott is considered tolerant to CI, but information about its response to low temperatures is scarce because of its recent commercialization. Under Mediterranean growing conditions, the fruit of Nadorcott was tolerant to CI, developing only minor symptoms in a low percentage of fruits even after prolonged exposure to cold (Figure 1 and Figure 2C). In agreement with our results, peel quality of Nadorcott fruit stored at 5 °C, and even lower temperatures (~0°), remained in good quality throughout storage, developing only slight pitting at the end of storage [46]. Fruit bagging before the natural development of peel color had a differential effect on the incidence of CI in each mandarin cultivar during cold storage (Figure 2A–C). In the CI-sensitive Fortune and Nova, chilling symptoms were delayed, whereas in the CI-tolerant Nadorcott they were enhanced (Figure 2A–C). However, the development of CI in sensitive cultivars was still high, and only a moderate reduction was observed after 8 weeks of storage. Furthermore, the effect of fruit bagging on CI, in Fortune and Nova, was not associated with an enhanced peel pigmentation at harvest and during postharvest storage (Figure 2D,E), in contrast to previous results in Star Ruby grapefruit in which the peel of shaded fruits turned red [5]. These results suggest that light avoidance has a differential effect on peel pigmentation in grapefruit and mandarin fruit, and also in the response of fruit to postharvest cold storage.

The CI tolerance in the red peel of the Star Ruby grapefruit has been directly related to carotenoid accumulation [5,21]. It was hypothesized that high TCC or specific individual carotenoids in the peel of mandarins could be related to the different susceptibility to CI. To explore this hypothesis, the profile and content of carotenoids were analyzed in the flavedo of the selected mandarins. TCC and carotenoid composition were markedly different between NC fruit of the three cultivars (Table 1, Table 2 and Table 3). At harvest, Nadorcott fruit accumulated the highest concentration of carotenoids (~580 µg g^−1^ FW), followed by Nova (~445 µg g^−1^ FW) and Fortune (~185 µg g^−1^ FW), in an inverse relationship with their susceptibility to CI (Figure 2A–C). Noteworthy is the extremely high TCC in the flavedo of Nadorcott mandarin, in accordance with the intense external orange coloration, making it one of the richest sources of carotenoids described thus far for citrus fruit [47,48]. The qualitative carotenoid composition was similar among cultivars and displayed the characteristic profile found in mandarin peel [10,11]. Levels of β-citraurin, which provides intense orange-reddish color to the peel [49,50,51], were similar among cultivars. The concentration of β-cryptoxanthin, a carotenoid, which also highly influences the characteristic intense orange color of mandarin peel [11,52], was about 2.5 and 20 times higher in Nadorcott than in Nova and Fortune, respectively (Table 1, Table 2 and Table 3). Thus, the combination of β-citraurin and β-cryptoxanthin contents in Nadorcott could explain the higher color index in this mandarin in comparison to the other cultivars. Moreover, β-citraurin levels in Fortune were high and represented about 25% of total carotenoids, whereas it accounted for 8% in Nova, which could explain why the color index was comparable between both cultivars when TCC was markedly lower in Fortune. High levels of this apocarotenoid have been previously reported in Fortune and related to a higher expression of the carotenoid-cleavage dioxygenase gene involved in β-citraurin synthesis [49]. Regarding the influence of carotenoids contents on CI, a strong negative correlation was found between TCC at harvest and CI index after prolonged cold storage (Table 4). Moreover, β-cryptoxanthin and violaxanthin contents highly influenced CI (Table 4), while other carotenoids (data not shown) and β-citraurin were not significantly correlated with CI (Table 4), indicating that not all carotenoids seem to play a role in the response of the fruit to cold stress.

Light avoidance has been shown to modify carotenoid content in citrus fruit [14,17], and we have used this effect to investigate the relationship between carotenoids and CI in mandarin fruits. Studies in Satsuma and Clemenules mandarin have reported that fruit exposed to lower sunlight, either grown inside of the tree canopy or bagged, accumulated lower concentrations of carotenoids compared to fruit grown under normal light incidence [4,7,14,45]. In Nadorcott and Nova mandarin, fruit bagging reduced TCC and the concentration of almost all carotenoids at harvest, but not the contents of the apocarotenoid β-citraurin (Table 2 and Table 3). The reduction of carotenoids in dark-grown citrus fruit has been associated with a down-regulation of main genes involved in carotenoid biosynthesis, however, the expression of the carotenoid-cleavage dioxygenase involved in β-citraurin biosynthetic was not affected by light conditions, which may explain the absence of an effect on this apocarotenoid [14]. Interestingly, during cold storage, the levels of β-citraurin remained high (>20 μg g^−1^ FW) in C fruits of the three mandarin cultivars, which may explain the maintenance of color index in these fruits (Table 1, Table 2 and Table 3). It is noteworthy that in Fortune, the effect of light avoidance on carotenoids was different from that occurring in the other two cultivars (Table 1). In dark-grown Fortune fruit, most of the carotenoids were similar to NC fruit, suggesting that Fortune fruit has a different regulatory mechanism of carotenoid biosynthesis and accumulation to the other mandarin cultivars, which may delineate an altered response to light avoidance.

The effect of storage at low temperatures (<5 °C) on peel color and carotenoids in mandarin cultivars has not been extensively investigated and seems to be highly dependent on the cultivar and fruit ripening stage at harvest [2]. Cold storage in Or and Odem mandarins has been reported to reduce peel coloration [53], while in Satsuma, a slight increase in carotenoid content has been reported [54]. It is interesting to note that, at the end of storage (8 weeks), TCC and specific individual carotenoids (as phytoene, phytofluene, and violaxanthin) in the peel of the three mandarins showed a differential response in accordance with their respective changes in the CI index (Figure 2A–C; Table 1, Table 2 and Table 3). In the fruit of the CI-sensitive Fortune, an important increase (38%) in TCC was observed in highly damaged NC fruit after 8 weeks of storage, which was reduced (7%) by bagging associated with a delay in the development of CI. No relevant changes were detected in fruit with a moderate CI index, such as Nova, and a decrease was detected in CI-tolerant Nadorcott fruit. These results suggest that the increase of carotenoids in injured mandarin may be part of the fruit’s response to cope against cold-damage. This, together with the high and inverse correlation between TCC and CI (Table 4), suggests that fruit of varieties or from environmental conditions with large carotenoid content at harvest may have better ability/tolerance to withstand cold damage during postharvest storage.

Vitamin C is a potent antioxidant metabolite in the peel of citrus fruit [27], and its water-soluble nature allows it to scavenge aqueous radicals efficiently [23]. The role of this compound in the development of CI in citrus fruit is still controversial, and while in certain species, a positive relation has been found [29], in others, higher contents were detected in CI-sensitive species [30,47]. In fruits of the three mandarin varieties studied in the current work, vitamin C content at harvest was directly related to CI sensitivity, and the levels did not substantially change during cold storage (Figure 3). Covering the fruit had a detrimental effect on vitamin C, as it reduced its concentration by over 60–70% with respect to light-exposed fruit (Figure 3). Similar results have been previously observed in the Navelina orange, Star Ruby grapefruit, and Satsuma and Nules Clementine mandarins, indicating that stimulation of AsA synthesis and accumulation by light is a general feature in *Citrus* species [7,25]. It has been proposed that in citrus fruit grown under dark or shaded conditions, the synthesis of vitamin C is impaired by a reduction of the expression of genes involved in the L-galacturonic acid pathway [25]—one of the four pathways regulating AsA biosynthesis in plants. A significant positive correlation (*r*^2^ = 0.82) was found between vitamin C content in fruit and CI index after cold storage (Table 4). This agrees with results in other *Citrus* species where it has been hypothesized that higher vitamin C content in sensitive fruit could be due to a higher demand for antioxidants as a defense mechanism [21,33], rather than an initial protective mechanism under cold stress. By contrast, in the fruit of other species, such as tomato, mango, or cucumber, a lower incidence of cold damage during storage has been associated with high levels of vitamin C [55,56,57]. Therefore, it is likely that the association between fruit vitamin C content and the susceptibility to develop CI is largely dependent on each species and the storage conditions.

Chilling damage in citrus fruit has been linked to harmful oxidative processes triggered by cold-induced ROS [2,3]. Thus, the antioxidant capacity of the fruit peel may be related to their cold tolerance. To explore this hypothesis, the antioxidant potential of the fruit peel of the three mandarins was assessed by three different methods (Figure 4 and Figure 5). The DPPH• and FRAP assays are based on electron transfer reactions and measure the ability of a hydrophilic extract to reduce a specific oxidant [58], while the SOAC assay assesses singlet oxygen absorption capacity of compounds present in a lipophilic extract and has been previously used to determine the carotenoid antioxidant ability in carotenoid-rich tissues, such as citrus fruit extracts [21,39]. In general, higher DPPH• and FRAP antioxidant activity were detected in extracts from CI-sensitive varieties than in extracts of the CI-tolerant Nadorcott (Figure 4), showing a significant positive correlation between CI and the DPPH• and FRAP antioxidant activity in the peel extracts of mandarin fruit (Figure 4; Table 4). Similarly, in chilling-sensitive fruit of Star Ruby grapefruit, higher DPPH•, ABTS, and ORAC antioxidant activity was detected [21]. In the three mandarin cultivars, the total antioxidant activity assessed by DPPH• and FRAP followed the same trend observed in vitamin C content (Figure 3), implying that these methods are appropriate to measure the contribution of hydrophilic compounds to the antioxidant system. Our correlation analysis also corroborated this conclusion as a strong positive correlation between vitamin C content and DPPH• and FRAP antioxidant activity was found (Table 4). By contrast, the relative SOAC values in the lipophilic extracts of the mandarins followed the same pattern as TCC and, interestingly, were negatively correlated with CI sensitivity (Figure 5; Table 4). In the peel of red grapefruit, a negative correlation was also reported between CI development and SOAC values, and also with TCC [21]. It was then proposed that high lycopene concentrations in CI-tolerant fruit [5] provides a superior ability to scavenge singlet oxygen, suggesting a protective role of this carotene in the development of CI in the peel of citrus fruit [21]. Mandarins are devoid of lycopene but contain significant amounts of other carotenoids, which have been associated with high antioxidant capacity in citrus [59]. Interestingly, the correlation analysis also showed a significant and strong positive relationship between β-cryptoxanthin and violaxanthin contents and SOAC values (Table 4). Therefore, these carotenoids, particularly abundant in the peel of mandarins, may play a protective role against cold stress by quenching singlet oxygen. Furthermore, these results support previous reports indicating that SOAC antioxidant capacity is a reliable and convenient system to assess the antioxidant capacity related to carotenoids as singlet oxygen quenchers, one of the main properties of these compounds [60] and a good indicator of CI tolerance.

Nonetheless, it is important to mention that the antioxidant lipophilic and hydrophilic capacity of citrus fruit extracts is provided by complex mixtures of many compounds, including carotenoids vitamin C and also tocopherols or polyphenols, among others [61]. Tocopherols and polyphenols are efficient ROS scavengers, and their relative abundance in extracts of citrus fruits may affect the antioxidant activities [62]. However, the particular contribution of tocopherols and polyphenols to the development of CI in citrus fruits is still unclear. In lemon, a lower concentration of tocopherols and flavonoids have been detected in CI-sensitive fruit [63]. In mandarins, an increase in phenylamonia-lyase activity, the rate-controlling enzyme of the phenylpropanoids pathway, has been associated with the development of CI during cold storage [64,65]. Regarding the enzymes scavenging active oxygen species, it has been suggested that catalase activity may be involved in the induction of chilling injury tolerance in mandarin and red grapefruit [21,66]. Then, it is likely that the antioxidant activity of the extracts used in the current study may be due to the presence not only of carotenoids and ascorbic acid but also other lipo- and hydrosoluble antioxidant components [67,68]. In this work, we have focused our attention on the potential contribution of carotenoids and vitamin C to the chilling tolerance, which has not been yet explored in mandarins. Carotenoids have been suggested to be the first line of defense against singlet oxygen toxicity in plants [69,70]. Their efficiency in quenching singlet oxygen has been proved for different carotenoids, including violaxanthin and β-cryptoxanthin [70,71]. In particular, β-cryptoxanthin has a potent in vitro antioxidant capacity and can also act as a scavenger of free radicals, preventing the oxidative damage of biomolecules and protecting against oxidative stress in in vivo systems [72,73,74]. Moreover, an elevated concentration of this carotenoid in orange juice samples was associated with a higher antioxidant capacity in cultivars not necessarily rich in TCC [75]. Therefore, considering the antioxidant properties of carotenoids and the results of the correlation analysis of the current work (Table 4), it is envisaged that β-cryptoxanthin and violaxanthin are major contributors to the SOAC capacity and to the CI susceptibility in mandarin fruits since highly significant positive and negative relationships, respectively, between these parameters were established.

## 5. Conclusions

Fruit of three mandarin cultivars with different susceptibility to develop CI (Fortune > Nova > Nadorcott) during cold storage were selected to investigate the potential relationship between CI, carotenoids, vitamin C, and antioxidant activity/capacity in hydrophilic and lipophilic peel extracts. Carotenoids and vitamin C content in the peel was manipulated by pre-harvest fruit bagging of the fruits. A strong negative correlation between TCC at harvest, specifically of β-cryptoxanthin and violaxanthin, and CI index at the end of the storage period was found, while vitamin C content was positively correlated with CI. The three methods used for the determination of antioxidant capacity showed that DPPH• and FRAP methods reflect the contribution of vitamin C to the antioxidant system, while SOAC correlated with TCC. Collectively, the antioxidant capacity of carotenoids at harvest, as efficient singlet oxygen quenchers, suggest a protective role for carotenoids, specifically β-cryptoxanthin and violaxanthin, against the development of CI in mandarin fruit during cold storage while the correlation analysis indicates that vitamin C is not likely playing a critical role.

## Figures and Tables

**Table 1:** Individual and total carotenoids (µg g^−1^ FW) detected in the peel of non-covered (NC) and covered (C) fruit of Fortune mandarin at harvest (0) and after 3 and 8 weeks of storage at 2 °C.

Carotenoids (µg g^−1^ FW)	FORTUNE																							
	0								3								8							
	NC				C				NC				C				NC				C			
Phytoene	6.34	±	0.40	**c**	17.02	±	2.53 **	**A**	12.61	±	2.09 *	**b**	7.64	±	0.20	**B**	19.62	±	1.24 **	**a**	5.09	±	2.20	**B**
Phytofluene	1.95	±	0.28	**a**	4.31	±	1.03 *	**A**	1.51	±	0.19	**a**	1.44	±	0.17	**B**	2.59	±	0.87	**a**	1.00	±	0.23	**B**
β-cryptoxanthin	3.44	±	1.02	**a**	2.23	±	0.30	**A**	3.56	±	2.27	**a**	5.29	±	2.63	**A**	1.96	±	0.43	**a**	8.23	±	3.19 *	**A**
β-citraurin	41.83	±	8.39	**a**	29.79	±	5.48	**A**	31.68	±	4.79	**a**	23.06	±	1.23	**A**	39.89	±	0.50 **	**a**	24.50	±	3.16	**A**
Antheraxanthin	14.45	±	1.34	**b**	14.25	±	1.13	**B**	24.49	±	1.64 **	**a**	7.91	±	0.24	**C**	31.14	±	2.61	**a**	24.74	±	1.69	**B**
Luteoxanthin	2.74	±	0.68	**b**	2.61	±	0.25	**B**	2.55	±	0.11 *	**b**	1.87	±	0.22	**B**	4.75	±	0.10	**a**	5.73	±	0.98	**A**
Violaxanthin	99.37	±	2.61	**a**	86.56	±	8.96	**A**	100.35	±	6.97	**a**	97.50	±	14.53	**A**	119.28	±	4.17	**a**	120.79	±	2.84	**A**
Lutein	0.58	±	0.34	**a**	0.88	±	0.40	**A**	1.19	±	0.01	**a**	nd				2.07	±	0.57	**a**	1.07	±	0.20	**A**
Other β.β-xanthophylls	12.99	±	2.58	**b**	25.62	±	5.65 *	**A**	23.66	±	3.24 *	**a**	8.96	±	4.39	**A**	29.52	±	0.96 **	**a**	10.05	±	3.82	**A**
**Total**	**185.90**	**±**	**17.86**	**b**	**189.53**	**±**	**13.67**	**A**	**204.03**	**±**	**15.33** *	**ab**	**157.28**	**±**	**14.24**	**A**	**256.32**	**±**	**9.12** **	**a**	**201.88**	**±**	**3.75**	**A**

Asterisks indicate significant differences between NC and C fruit for each cultivar and sample date (** *p* ≤ 0.05; * *p* ≤ 0.10, *t*-test). Bold lowercase letters indicate significant differences between dates in NC fruit and bold capital letters indicate significant differences between dates in C fruit (*p* ≤ 0.05, Tukey test). nd: non-detected.

**Table 2:** Individual and total carotenoids (µg g^−1^ FW) detected in the peel of non-covered (NC) and covered (C) fruit of Nova mandarin at harvest (0) and after 3 and 8 weeks of storage at 2 °C.

Carotenoids (µg g^−1^ FW)	NOVA																							
	0								3								8							
	NC				C				NC				C				NC				C			
Phytoene	98.12	±	8.60 **	**a**	41.19	±	4.01	**C**	120.23	±	4.37 **	**a**	81.11	±	4.83	**A**	103.43	±	9.12 **	**a**	59.65	±	3.30	**B**
Phytofluene	24.17	±	2.40 **	**a**	12.71	±	0.76	**C**	36.26	±	0.48 **	**a**	28.34	±	1.40	**A**	32.32	±	4.38 *	**a**	18.56	±	1.18	**B**
β-cryptoxanthin	25.36	±	1.43 *	**a**	19.47	±	1.43	**A**	44.54	±	0.95	**b**	27.24	±	14.12	**A**	24.09	±	4.18 *	**b**	12.54	±	2.02	**A**
β-citraurin	37.77	±	2.97	**a**	58.69	±	5.68 **	**A**	23.32	±	0.55	**b**	39.22	±	11.51	**A**	36.26	±	0.59	**a**	36.29	±	0.43	**A**
Antheraxanthin	29.70	±	2.15 **	**a**	8.22	±	2.27	**B**	34.90	±	4.01 *	**a**	22.85	±	0.86	**A**	34.93	±	0.80 **	**a**	17.55	±	0.46	**A**
Luteoxanthin	5.35	±	2.64	**a**	2.44	±	0.02	**A**	4.15	±	0.19 **	**a**	1.83	±	0.40	**A**	5.65	±	0.25 **	**a**	2.41	±	0.39	**A**
Violaxanthin	169.09	±	4.00 **	**a**	133.12	±	0.56	**A**	159.17	±	6.42 **	**a**	116.84	±	8.21	**A**	168.07	±	1.13 **	**a**	134.73	±	2.20	**A**
Lutein	7.56	±	0.09 *	**a**	2.59	±	1.95	**A**	9.06	±	1.10 **	**a**	4.11	±	0.96	**A**	7.67	±	1.03 *	**a**	3.22	±	0.03	**A**
Other β.β-xanthophylls	39.52	±	10.13	**a**	25.00	±	14.30	**A**	32.96	±	6.58 **	**a**	7.38	±	0.66	**A**	38.09	±	5.56 *	**a**	14.83	±	6.96	**A**
**Total**	**444.36**	**±**	**1.59** **	**a**	**308.85**	**±**	**17.79**	**A**	**478.06**	**±**	**16.59** **	**a**	**338.44**	**±**	**41.35**	**A**	**463.61**	**±**	**15.52** **	**a**	**304.30**	**±**	**9.26**	**A**

Asterisks indicate significant differences between NC and C fruit for each cultivar and sample date (** *p* ≤ 0.05; * *p* ≤ 0.10, *t*-test). Bold lowercase letters indicate significant differences between dates in NC fruit and bold capital letters indicate significant differences between dates in C fruit (*p* ≤ 0.05, Tukey test).

**Table 3:** Individual and total carotenoids (µg g^−1^ FW) detected in the peel of non-covered (NC) and covered (C) fruit of Nadorcott mandarin at harvest (0) and after 3 and 8 weeks of storage at 2 °C.

Carotenoids (µg g^−1^ FW)	NADORCOTT																							
	0								3								8							
	NC				C				NC				C				NC				C			
Phytoene	59.90	±	1.18 **	**a**	29.75	±	1.07	**A**	48.06	±	0.21 **	**b**	24.43	±	0.84	**A**	21.06	±	3.87 *	**c**	12.29	±	1.77	**B**
Phytofluene	17.30	±	2.21 **	**a**	7.70	±	0.30	**A**	12.88	±	1.44 **	**a**	3.69	±	0.63	**B**	11.28	±	0.36 **	**a**	5.51	±	0.49	**B**
β-cryptoxanthin	61.46	±	8.05	**ab**	68.87	±	16.53	**A**	84.87	±	5.54 **	**a**	66.98	±	0.83	**A**	54.15	±	4.73	**b**	68.38	±	2.46 *	**A**
β-citraurin	56.75	±	9.55	**a**	46.22	±	6.36	**A**	32.69	±	1.26	**a**	30.13	±	5.38	**A**	48.52	±	5.48 **	**a**	31.15	±	1.02	**A**
Antheraxanthin	42.74	±	2.15 **	**b**	26.37	±	2.22	**B**	58.21	±	1.70 **	**a**	42.56	±	0.64	**A**	50.96	±	4.52 **	**ab**	33.08	±	1.59	**B**
Luteoxanthin	7.54	±	3.36	**a**	2.85	±	1.00	**A**	3.87	±	2.97	**a**	5.41	±	0.63	**A**	5.06	±	1.37	**a**	2.05	±	0.15	**A**
Violaxanthin	264.06	±	16.51 **	**a**	209.95	±	2.14	**A**	242.69	±	3.51 **	**ab**	225.78	±	1.06	**A**	218.18	±	3.71	**b**	202.19	±	19.83	**A**
Lutein	9.82	±	1.82	**a**	8.30	±	0.65	**B**	17.41	±	2.54	**a**	13.33	±	0.03	**A**	11.23	±	2.28	**a**	12.19	±	1.19	**A**
Other β.β-xanthophylls	51.10	±	5.83 **	**a**	18.72	±	2.21	**A**	54.06	±	9.67 **	**a**	13.65	±	0.94	**AB**	34.14	±	5.62 *	**a**	12.58	±	0.08	**B**
**Total**	**582.20**	**±**	**22.85** **	**a**	**424.99**	**±**	**16.80**	**AB**	**565.05**	**±**	**9.10** **	**a**	**430.16**	**±**	**2.50**	**A**	**479.82**	**±**	**14.39** **	**b**	**381.69**	**±**	**25.44**	**B**

Asterisks indicate significant differences between NC and C fruit for each cultivar and sample date (** *p* ≤ 0.05; * *p* ≤ 0.10, *t*-test). Bold lowercase letters indicate significant differences between dates in NC fruit and bold capital letters indicate significant differences between dates in C fruit (*p* ≤ 0.05, Tukey test).

**Table 4:** Pearson’s correlation coefficients (*r*^2^) among CI at the end of storage (8 weeks) and total carotenoid content (TCC), individual carotenoids (β-cryptoxanthin, β-citraurin, and Violaxanthin), vitamin C and antioxidant capacity/activity (SOAC, DPPH• and FRAP) at harvest.

	CI	TCC	β-Cryptoxanthin	β-Citraurin	Violaxanthin	Vitamin C	SOAC	DPPH•	FRAP
CI	1	−0.81 *	−0.82 *	−0.74	−0.85 *	0.82 *	−0.88 *	0.89 *	0.81 *
TCC	−0.81 *	1	0.85 *	0.52	0.97 *	−0.62	0.96 *	−0.52	−0.41
β-cryptoxanthin	−0.82 *	0.85 *	1	0.51	0.93 *	−0.78	0.92 *	−0.73	−0.71
β-citraurin	−0.74	0.52	0.51	1	0.57	−0.51	0.55	−0.75	−0.71
Violaxanthin	−0.85 *	0.97 *	0.93 *	0.57	1	−0.64	0.99 *	−0.62	−0.57
Vitamin C	0.82 *	−0.62	−0.78	−0.51	−0.64	1	−0.66	0.87 *	0.75
SOAC	−0.88 *	0.96 *	0.92 *	0.55	0.99 *	−0.66	1	−0.65	−0.60
DPPH•	0.89 *	−0.52	−0.73	−0.75	−0.62	0.87 *	−0.65	1	0.96 *
FRAP	0.81 *	−0.41	−0.71	−0.71	−0.57	0.75	−0.60	0.96 *	1

Asterisk indicates significant Pearson’s correlation coefficient at level *p* ≤ 0.05.

## Supplementary Materials

The following are available online at https://www.mdpi.com/2076-3921/9/12/1296/s1, Figure S1: Pictures of the scale used to rate the severity of CI symptoms during postharvest cold storage; Figure S2: MaxPlot chromatogram of the saponified carotenoid extract of the Nadorcott flavedo at harvest; Figure S3: Chromatogram at 248 nm showing ascorbic acid peak; Figure S4: Pictures illustrating CI symptoms severity and magnification of CI development in fruit of Fortune, Nova, and Nadorcott; Table S1: Carotenoid limits of detection and working linear range; Table S2: Chromatographic and spectroscopic characteristics of carotenoids found in the flavedo of the three mandarin cultivars.
