# Supplementary material for: Carotenoids, Vitamin C, and Antioxidant Capacity in the Peel of Mandarin Fruit in Relation to the Susceptibility to Chilling Injury during Postharvest Cold Storage
Source: Antioxidants (Basel). 2020 Dec 17;9(12):1296. doi: 10.3390/antiox9121296 (PMC7766470; doi:10.3390/antiox9121296)
Supplement: Supplementary file 1 [file antioxidants-09-01296-s001.pdf]

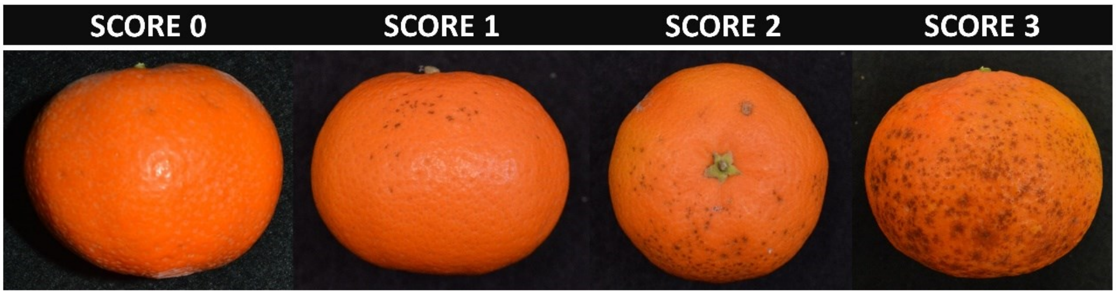

Figure S1. Pictures of the scale used to rate the severity of CI symptoms during postharvest cold storage. Score 0: no pitting; Score 1: small pits covering <25 % of the fruit surface; Score 2: larger and darker pits covering between 25-50 % of the surface; and Score 3: severe damage covering >50 % of the surface.

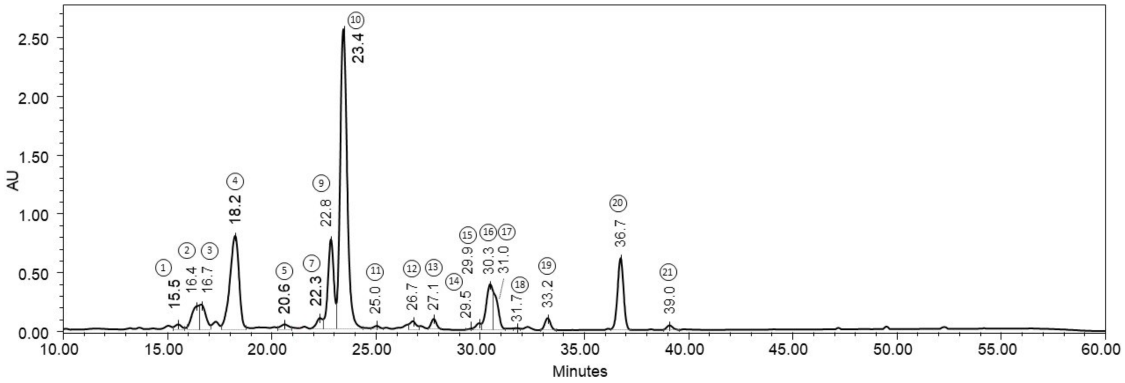

Figure S2. MaxPlot chromatogram of the saponified carotenoid extract of the Nadorcott flavedo at harvest. Peaks correspond to (1) NI (327(Z), 411,433,461); (2) NI (327(Z), 405,428,456); (3) NI (328(Z), 409,431,458); (4) All-*E*-violaxanthin; (5) Luteoxanthin; (7) NI (415,438,469); (9) β-citraurin; (10) 9-*Z*-violaxanthin; (11) Luteoxanthin isomer; (12) Lutein; (13) NI (410,435,464); (14) Zeaxanthin; (15) Mutatoxanthin; (16) Antheraxanthin; (17) 15-*Z*-Phytoene; (18) NI (sh,446,470); (19) Phytofluene; (20) β-cryptoxanthin; and (21) ζ-carotene. Missing peaks in the chromatogram but present in Table S1 are carotenoids not present in this specific sample (i.e. peak 6, 8 and 22). Peaks not numbered in the chromatogram do not show the absorbance carotenoid-type spectrum.

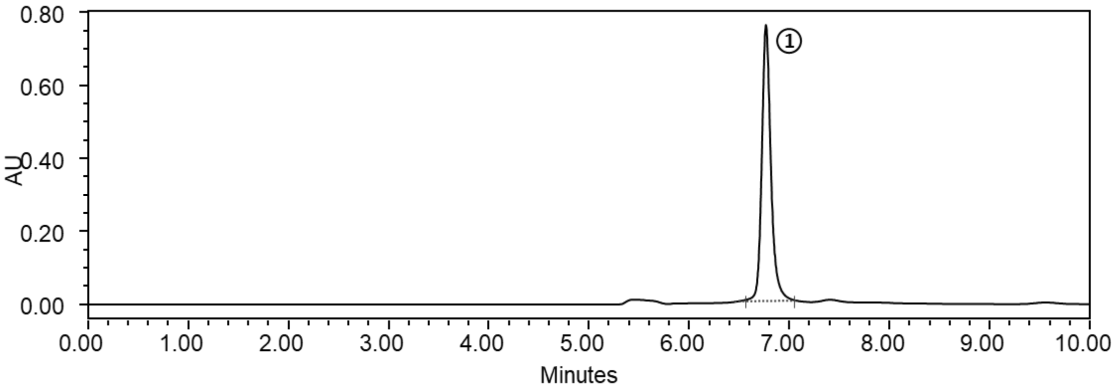

Figure S3. HPLC chromatogram at 248 nm showing ascorbic acid peak (1).

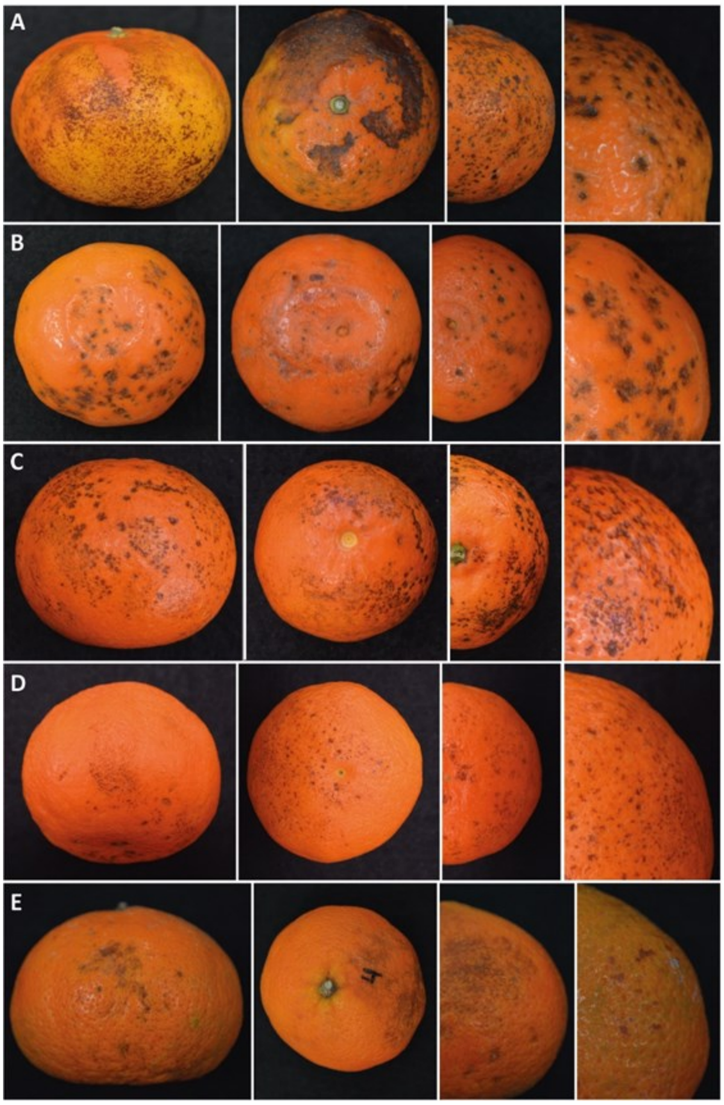

Figure S4. Pictures illustrating CI symptoms severity and magnification of CI development in fruit of Fortune non-covered (**A**) and covered (**B**), Nova non-covered (**C**) and covered (**D**), and covered fruit of Nadorcott (**E**).

Table S1. Carotenoid quantification parameters. Carotenoids were quantified by HPLC-PAD by integration of the peaks in MaxPlot chromatograms (Waters Empower Software). Violaxanthin isomers, luteoxanthin and other xanthophylls not specified were quantified using (all-*E*)-violaxanthin (CaroteNature). β-Citraurin was quantified using β-apo-8′-carotenal (Extrasynthese). Standards of lutein, zeaxanthin and β-cryptoxanthin were purchase from Extrasynthese, and antheraxanthin from CaroteNature. Phytoene and phytofluene were HPLC-purified from sweet orange Pinalate extracts [36] and used as standards for quantification. The concentrations of (*Z*)-isomers were determined using the corresponding (all-*E*) xanthophylls. The limits of detection (LOD) and working linear range are indicated for lutein, violaxanthin, antheraxanthin, zeaxanthin, β-cryptoxanthin, phytoene, phytofluene, β-apo-8′-carotenal.

Carotenoid	LOD[a]	Working linear range (mg/L)
Phytoene	25	1.52 to 350
Phytofluene	20	1.21 to 200
β-Cryptoxanthin	10	0.55 to 99
Zeaxanthin	14	0.72 to 85
Antheraxanthin	11	0.54 to 102
Violaxanthin	14	0.76 to 98
Lutein	15	0.21 to 120
β-apo-8′-carotenal	14	0.82 to 60

[a] The LOD was calculated as the amount (in ng) load on column at which the signal level of the carotenoid reaches at least 3 times the signal noise of the baseline.

Table S2. Chromatographic and spectroscopic characteristics of carotenoids found in the flavedo of the three mandarin cultivars.

Peak	Retention time (min)	Carotenoid [a]	UV-Vis absorption maxima (nm)	D_B/D_{II} [b]
**1**	15.5	NI	327(Z), 411, 433, 461	0.31
**2**	16.4	NI	327(Z), 405, 428, 456	0.26
**3**	16.7	NI	328(Z), 409, 431, 458	0.34
**4**	18.2	*All-*E*-violaxanthin	415, 438, 468	
**5**	20.6	Luteoxanthin	396, 421, 448	
**6**	21.2	NI [F, N]	sh, 425, 450	
**7**	22.3	NI	415, 438, 469	
**8**	22.6	NI [F]	409, 431, 459	
**9**	22.8	β-citraurin	459	
**10**	23.4	*9-*Z*-violaxanthin	328(Z), 412, 436, 464	0.06
**11**	25.0	Luteoxanthin isomer	398, 416, 443	
**12**	26.7	*Lutein	sh, 443, 472	
**13**	27.1	NI	410, 435, 464	
**14**	29.5	*Zeaxanthin	sh, 450, 475	
**15**	29.9	Mutatoxanthin	sh, 427, 450	
**16**	30.3	*Antheraxanthin	329(Z), sh, 441, 469	0.06
**17**	31.0	*15-*Z*-Phytoene	285	
**18**	31.7	NI [N, Na]	sh, 446, 470	
**19**	33.2	*Phytofluene	331,346,364	
**20**	36.7	*β-cryptoxanthin	423,450,479	
**21**	39.0	*ζ-carotene	378, 399 ,424	
**22**	43.5	NI [N, Na]	sh, 450, 480	

[a] *, identified using authentic standards; NI, not identified; [F], only in Fortune; [N], only in Nova; [Na], only in Nadorcott. [b] Intensity of Z peak as ratio D_B/D_{II}.
